# Supplementary material for: Magnesium Hydroxide Nanoparticles Inhibit the Biofilm Formation of Cariogenic Microorganisms
Source: Nanomaterials (Basel). 2023 Feb 25;13(5):864. doi: 10.3390/nano13050864 (PMC10005196; doi:10.3390/nano13050864)
Supplement: Supplementary file 1 [file nanomaterials-13-00864-s001.zip › nanomaterials-2194031-supplementary.pdf]

nanomaterials

Supplementary Materials

Magnesium Hydroxide Nanoparticles Inhibit the Biofilm Formation of Cariogenic Microorganisms

Kentaro Okamoto [1,2], Daisuke Kudo [3], Dao Nguyen Duy Phuong [3], Yoshihito Iwamoto [3], Koji Watanabe [2], Yoshie Yoshioka [1], Wataru Ariyoshi [1] and Ryota Yamasaki [1,4,*]

[1] Division of Infections and Molecular Biology, Department of Health Promotion, Kyushu Dental University, Kitakyushu, Fukuoka 803-8580, Japan
[2] Division of Developmental Stomatognathic Function Science, Department of Health Promotion, Kyushu Dental University, Kitakyushu, Fukuoka 803-8580, Japan;
[3] SETOLAS Holdings Inc., Hayashida-cho, Sakaide, Kagawa 762-0012, Japan;
[4] Collaborative Research Centre for Green Materials on Environmental Technology, Kyushu Institute of Technology, 1-1 Sensui-chou, Tobata-ku, Kitakyushu, Fukuoka 804-8550, Japan
* Correspondence: r18yamasaki@fa.kyu-dent.ac.jp; Tel.: +81-93-285-3051

Keywords: magnesium hydroxide nanoparticle; dental caries; cariogenic microorganisms; biofilm inhibition

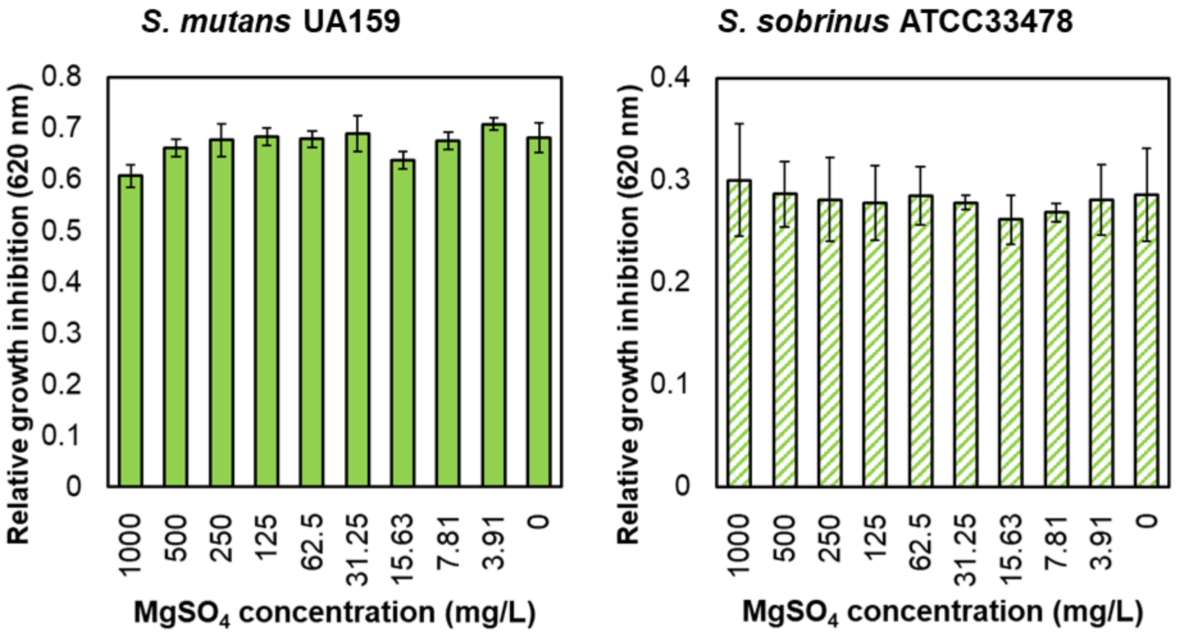

Figure S1. Growth inhibitory effects of 1,000 mg/L- 0 mg/L $MgSO_4$ (a 2-fold serial dilution was applied). Left indicates *S. mutans* and right indicates *S. sobrinus*. Error bars indicate standard deviations of at least three experiments. Student's *t*-test were used to compare the two groups (* indicates a *p*-value < 0.05 and ** indicates a *p*-value < 0.01).

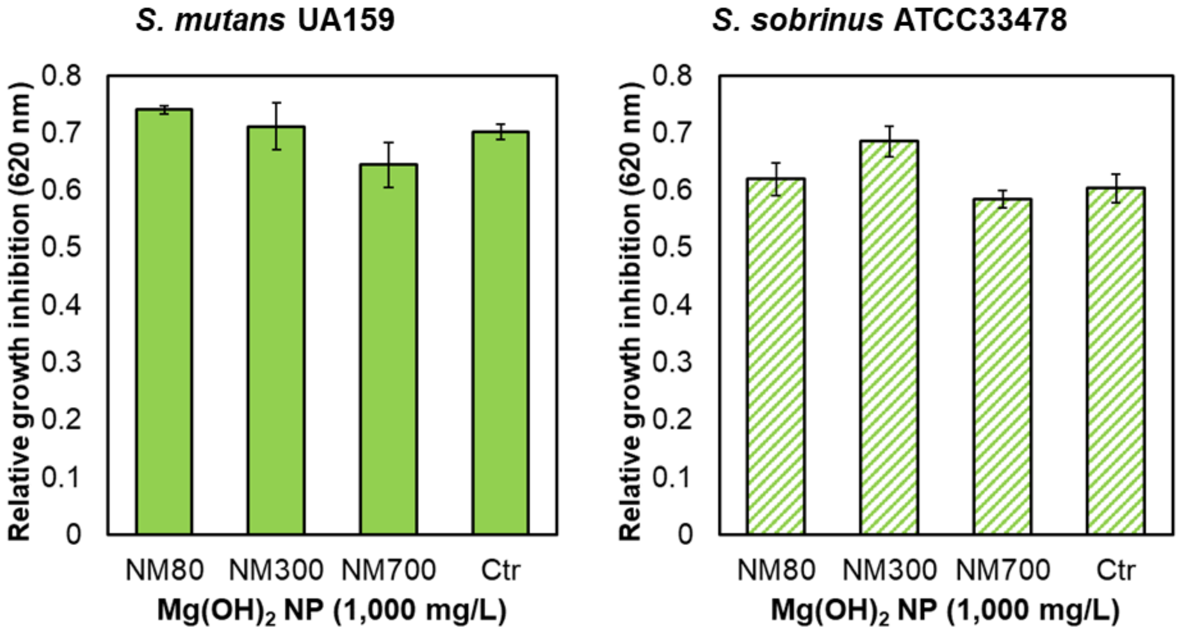

Figure S2. Growth inhibitory effects by 1,000 mg/L $Mg(OH)_2$ NP pre-treatment. Left indicates *S. mutans* and right indicates *S. sobrinus*. Error bars indicate standard deviations of at least three experiments.

Table S1. Growth inhibition data as described in Figure 2.

	Conc. (mg/L)	1000	500	250	125	62.5	31.25	15.63	7.81	3.91	0
S. mutans	NM80	0.55 ± 0.02	0.63 ±0.04	0.65 ± 0.01	0.64 ± 0.02	0.63 ± 0.01	0.63 ± 0.02	0.58 ± 0.01	0.60 ± 0.01	0.60 ± 0.02	0.607 ± 0.006
	NM300	0.585 ± 0.008	0.584 ± 0.007	0.66 ± 0.02	0.66 ± 0.01	0.649 ± 0.004	0.64 ± 0.02	0.62 ± 0.02	0.63 ± 0.02	0.62 ± 0.01	0.60 ± 0.01
	NM700	0.599 ± 0.008	0.59 ± 0.01	0.613 ± 0.008	0.61 ± 0.03	0.6 1± 0.03	0.62 ± 0.03	0.62 ± 0.02	0.62 ± 0.01	0.62 ± 0.01	0.600 ± 0.005
S. sobrinus	NM80	0.60 ± 0.03	0.52 ± 0.03	0.53 ± 0.02	0.53 ± 0.04	0.53 ± 0.03	0.52 ± 0.01	0.50 ± 0.07	0.55 ± 0.04	0.54 ± 0.07	0.54 ± 0.08
	NM300	0.62 ± 0.03	0.50 ± 0.01	0.53 ± 0.03	0.53 ± 0.04	0.5 2± 0.04	0.54 ± 0.02	0.47 ± 0.04	0.51 ± 0.03	0.51 ± 0.03	0.56 ± 0.01
	NM700	0.63 ± 0.02	0.61 ± 0.02	0.56 ± 0.03	0.49 ± 0.05	0.5 ± 0.1	0.53 ± 0.02	0.51 ± 0.03	0.49 ± 0.06	0.53 ± 0.03	0.54 ± 0.02

Table S2. Biofilm inhibition data as described in Figure 2.

	Conc. (mg/L)	1000	500	250	125	62.5	31.25	15.63	7.81	3.91	0
S. mutans	NM80	0.55 ± 0.06	0.9 ± 0.1	0.85 ± 0.07	1.09 ± 0.07	1.17 ± 0.04	1.2 ± 0.1	1.15 ± 0.09	1.25 ± 0.09	1.19 ± 0.09	1.19 ± 0.04
	NM300	0.3 ± 0.1	0.690 ± 0.008	1.05 ± 0.09	1.22 ± 0.07	1.40 ± 0.08	1.49 ± 0.09	1.42 ± 0.03	1.5 ± 0.1	1.46 ± 0.03	1.33 ± 0.03
	NM700	0.18 ± 0.02	0.61 ± 0.06	1.04 ± 0.02	1.4 ± 0.1	1.49 ± 0.06	1.5 ± 0.2	1.4 ± 0.1	1.4 ± 0.2	1.43 ± 0.07	1.4 ± 0.1
S. sobrinus	NM80	0.76 ± 0.06	1.1 ± 0.2	2.2 ± 0.4	2.2 ± 0.1	2.32 ± 0.09	2.3 ± 0.2	2.2 ± 0.2	2.3 ± 0.2	2.6 ± 0.5	2.2 ± 0.2
	NM300	0.74 ± 0.02	1.6 ± 0.1	2.6 ± 0.3	2.6 ± 0.3	2.5 ± 0.4	2.5 ± 0.4	2.5 ± 0.2	2.7 ± 0.3	2.7 ± 0.4	2.2 ± 0.2
	NM700	0.552 ± 0.002	0.96 ± 0.07	2.6 ± 0.4	2.9 ± 0.5	2.8 ± 0.7	2.89 ± 0.04	2.9 ± 0.4	2.9 ± 0.3	2.7 ± 0.3	2.5 ± 0.1

Table S3. Biofilm inhibition data by pH effect as described in Figure 3A. Each $Mg(OH)_2$ NP was concentrated at 1,000 mg/L.

	**NM80**	**NM300**	**NM700**	**pH8.5**	**pH=7**
S. mutans	0.55 ± 0.06	0.3 ± 0.1	0.18 ± 0.02	1.0 ± 0.2	1.31 ± 0.03
S. sobrinus	0.76 ± 0.06	0.74 ± 0.02	0.552 ± 0.002	1.7 ± 0.2	2.29 ± 0.07

Table S4. Biofilm inhibition data by magnesium ion ($MgSO_4$) effect as described in Figure 3B.

	1000	500	250	125	62.5	31.25	15.63	7.81	3.91	0
S. mutans	1.3 ± 0.1	1.25 ± 0.08	1.3 ± 0.1	1.4 ± 0.1	1.4 ± 0.2	1.2 ± 0.1	1.4 ± 0.1	1.4 ± 0.1	1.3 ± 0.2	1.2 ± 0.1
S. sobrinus	2.9 2± 0.09	2.92 ± 0.09	2.9 ± 0.1	2.9 ± 0.1	3.0 ± 0.2	3.0 ± 0.1	3.0 ± 0.2	3.0 ± 0.2	2.97 ± 0.06	2.3 ± 0.2

Table S5. Attachment inhibition data by pre-treating of each $Mg(OH)_2$ NP (1,000 mg/L) as described in Figure 4A.

	**NM80**	**NM300**	**NM700**	**Ctr**
S. mutans	1.5 ± 0.1	0.8 ± 0.1	0.9 ± 0.1	1.5 ± 0.1
S. sobrinus	1.9 ± 0.1	0.44 ± 0.04	0.47 ± 0.04	1.7 ± 0.5

Table S6. Biofilm dispersal data by treating with each $Mg(OH)_2$ NP (1,000 mg/L) as described in Figure 4B.

	NM80	NM300	NM700	Ctr
S. mutans	4.5 ± 0.9	3.0 ± 0.4	2.7 ± 0.2	1.6 ± 0.2
S. sobrinus	6.5 ± 0.5	3.4 ± 0.3	3.1 ± 0.6	1.4 ± 0.4
